# Exploring the molecular mechanism of Xiao Ji (*Cirsium setosum*) in treating bladder cancer using network pharmacology and molecular docking

**DOI:** 10.2478/abm-2025-0012

**Published:** 2025-04-30

**Authors:** Hui Yu, Yang Dong, Gui-cheng Zou, Yun-jie Yang, Meng-zhen Liu, Cong-hui Han

**Affiliations:** 1 Integrated Traditional Chinese and Western Medicine Clinical Medicine School,Nanjing University of Chinese Medicine, No. 138 Xianlin Avenue, Qixia District, Nanjing, Jiangsu 210023, China; 2 Department of Urology, Yantai Hospital of Traditional Chinese Medicine, Yantai, Shandong 264001, China; 3 Department of Urology, Xuzhou Central Hospital Affiliated to Nanjing University of Chinese Medicine, Xuzhou, Jiangsu 221006, China; 4 Department of Pelvic Floor Rehabilitation Center, The Huai’an Maternity and Child Clinical College of Xuzhou Medical University, Huai’an, Jiangsu 223002, China; 5 Department of Oncology, Zhumadian Central Hospital, Zhumadian, Henan 463000, China

**Keywords:** bladder cancer, molecular docking, network pharmacology, target prediction, Xiao Ji

## Abstract

**Background:**

In China, Xiao Ji decoction has been used to treat hematuria. However, pharmacological studies on its effects against bladder cancer (BC) remain limited.

**Objective:**

This study aims to explore the potential mechanisms of Xiao Ji in treating BC using network pharmacology and molecular docking.

**Methods:**

The active constituents of Xiao Ji and their corresponding molecular targets were identified through the utilization of the Traditional Chinese Medicine Systems Pharmacology Database. Genes associated with BC were screened by employing resources including the Online Mendelian Inheritance in Man (OMIM) and GeneCards databases. Furthermore, protein–protein interaction (PPI) networks and networks illustrating the connections between ingredients and their ingredient–target (I–T) were established. The related genes underwent gene ontology (GO) and Kyoto Encyclopedia of Genes and Genomes (KEGG) pathway enrichment analyses. Ultimately, molecular docking experiments were conducted to substantiate and reinforce the proposed hypotheses.

**Results:**

Four compounds were identified, along with 82 target genes that exhibited associations with BC. In the I–T network, quercetin exhibited the highest degree of association with multiple targets. Within the PPI network, interleukin (IL)IL-6, hypoxia inducible factor 1 subunit alpha (HIF1A), epidermal growth factor receptor (EGFR), and myelocytomatosis oncogene (MYC) were discerned as pivotal genes. The enrichment analysis of the critical genes led to the identification of 92 GO terms and 105 pathways. Furthermore, the results of molecular docking analyses revealed that the active compounds, including acacetin, sitosterol, and stigmasterol, exhibited strong binding affinities with IL-6, EGFR, and MYC.

**Conclusions:**

Xiao Ji acts on BC through multiple targets and pathways. This study elucidates the potential mechanisms of Xiao Ji in treating BC, providing new options for BC therapy.

Bladder cancer (BC) ranks among the most prevalent malignant neoplasms, with an estimated 614,298 new cases reported globally in the year 2022 (Source: Global Cancer Observatory), leading to significant economic burdens. Cigarette smoking stands as the foremost and pivotal risk factor associated with BC [1]. Painless hematuria represents the most prevalent symptom, and cystoscopy is unequivocally recognized as the gold standard for diagnosing BC [2]. Approximately 75% of BC cases are classified as non-muscle invasive bladder cancer (NMIBC), for which the primary treatment approach is typically transurethral resection of bladder tumor (TURBT) [3]. The progression rate of NMIBC to muscle-invasive bladder cancer (MIBC) is estimated to be approximately 15%–20% [4]. Following TURBT, intravesical therapy is recommended based on the risk classification. Bacillus Calmette–Guérin (BCG) immunotherapy is commonly employed for intermediate- and high-risk NMIBC. However, it is worth noting that despite its effectiveness, the recurrence rate remains notably high in these cases [5]. Immune checkpoint inhibitors are studied intensively in BCG refractory NMIBC [6], and pembrolizumab has been approved in the treatment of BCG failure NMIBC [7]. Recurrent high-risk NMIBC has a low survival rate and a high progression rate [8], and there remains a critical need for pharmaceutical agents that can effectively diminish the recurrence and progression rates of NMIBC. Removal of the entire bladder, known as radical cystectomy (RC), is often necessary for the treatment of MIBC. The metastasis rate is approximately 50% after RC [9], and perioperative cisplatinbased chemotherapy is suggested. Cisplatin-based chemotherapy has been the established standard for systemic therapy in the treatment of both MIBC and metastatic BC. This approach has been widely adopted over the past three decades [9]. Novel therapies such as targeted therapies and immune checkpoint inhibitors have shown promise in improving therapeutic outcomes for BC, but outcomes in the treatment of BC remain unsatisfactory [10]. There remains a need for the development of more effective agents and treatment strategies for BC.

Natural plants serve as an important source of therapeutic agents. In the realm of traditional Chinese medicine (TCM), *Cirsium setosum*, known as Xiao Ji, has been employed for centuries in the treatment of hematuria. Pharmacological research has demonstrated that compounds extracted from Xiao Ji possess tumor-suppressive properties, particularly in the case of ovarian cancer and colon cancer [11]. Painless hematuria is a primary symptom of BC and given the potential tumor-suppressive properties of compounds from Xiao Ji in other cancer types, investigating whether Xiao Ji has anti-BC properties, which is a worthwhile avenue of study. Research in this area could shed light on its potential therapeutic role in managing BC.

Network pharmacology is an effective strategy for exploring and identifying potential new drugs and therapeutic interventions. It leverages complex network analyses to understand the interactions between drugs, targets, and diseases, aiding in the identification of novel therapeutic candidates and the elucidation of their mechanisms of action. This approach has become increasingly valuable in modern drug discovery and development processes. Molecular docking is a computational process that involves the interaction of small molecules (ligands) with macromolecules (typically proteins) to predict and evaluate the binding affinity and orientation of the ligand within the binding site of the macromolecule. The docking score, which is a numerical value, is used to assess the strength of the interaction between the ligand and the macromolecule. In drug discovery, molecular docking is employed to identify potential drug candidates by evaluating the binding affinity of various compounds to a specific target protein. Compounds with high docking scores are considered promising candidates for further experimental testing and development as potential drugs [12]. Molecular docking offers a relatively fast and cost-effective alternative to experimental approaches [13]. The objective of this study was to employ a network pharmacology approach to explore the key active ingredients and potential anticancer mechanisms of Xiao Ji in the context of BC. Additionally, the findings from the network pharmacology analysis were further validated through molecular docking experiments. This integrated approach aimed to provide insights into the potential therapeutic properties of Xiao Ji against BC and to elucidate the underlying molecular mechanisms. The study’s methodology and workflow are visually depicted in Figure 1.

**Figure 1.**
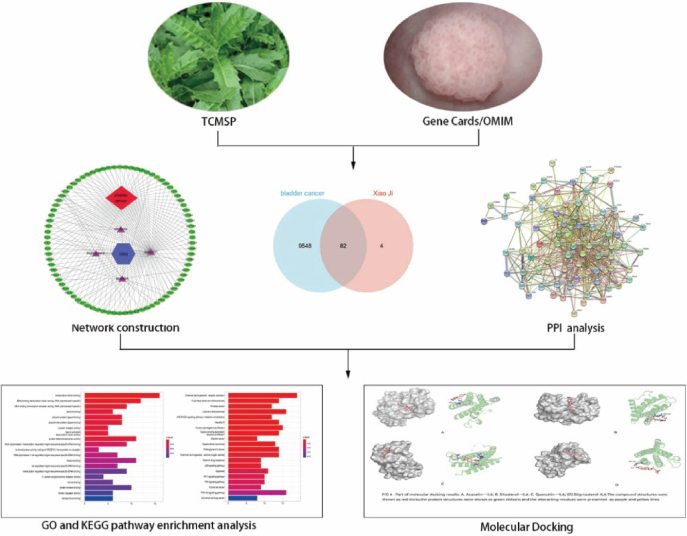
Research process. GO, gene ontology; KEGG, Kyoto Encyclopedia of Genes and Genomes; OMIM, Online Mendelian Inheritance in Man; PPI, protein–protein interaction; TCMSP, TCM systems pharmacology.

## Methods

### Screening the compounds of Xiao Ji

The compounds of Xiao Ji were identified utilizing TCM systems pharmacology (TCMSP) database (developed by Northwest A&F University, China). TCMSP is a Chinese herbal medicine database, which can provide relationships among herbs, target proteins, and diseases [14]. Based on a combination of previous literature and the guidelines provided by the TCMSP, compounds meeting the criteria of having an oral bioavailability (OB) of at least 30% and a drug-likeness (DL) score of ≥0.18 were chosen as candidate ingredients for further detailed analysis.

### Molecular target prediction

The targets associated with the selected compounds were obtained by inputting these compounds into the TCMSP database. The gene symbols corresponding to the identified targets were retrieved from the UniProt Knowledgebase (developed by the UniProt Consortium,https://www.uniprot.org).

### Identification of targets specific to BC

Genes associated with BC were obtained from the GeneCards database (developed by the Weizmann Institute of Science, Israel, https://www.genecards.org) and the Online Mendelian Inheritance in Man (OMIM) database (Online Mendelian Inheritance in Man, developed by Johns Hopkins University, Baltimore, MD, USA). Finally, we intersected the target genes of the active ingredients from Xiao Ji with the target genes associated with BC and selected the common genes as related genes of Xiao Ji for treating BC.

### Ingredient–target network construction

The corresponding relationships of disease, key targets, herbs, and active ingredients were constructed using Microsoft Excel to manipulate and organize data, followed by importing the data into Cytoscape 3.8.0 software (developed by the Cytoscape Consortium, Institute for Systems Biology, Seattle, WA, USA). The ingredient–target (I–T) interaction network was visualized using Cytoscape.

### Protein–protein interaction network construction

The identified target proteins were submitted to the STRING database (developed by the Swiss Institute of Bioinformatics, https://string-db.org) for the purpose of scrutinizing the interactions that transpire among these proteins, thereby facilitating the construction of a protein–protein interaction (PPI) network. The interaction data were entered into Cytoscape to facilitate the visualization of the PPI network.

### Gene ontology and Kyoto encyclopedia of genes and genomes enrichment analysis

Gene ontology (GO) and Kyoto encyclopedia of genes and genomes (KEGG) enrichment analyses represent efficacious methodologies for the prediction and analysis of the functional roles of pivotal genes. We conducted GO and KEGG pathway enrichment analyses using R packages, namely “DOSE” (Guangchuang Yu, Southern Medical University), “clusterProfiler” (Guangchuang Yu, Southern Medical University), and “pathview” (Weijun Luo). The significance threshold applied for both analyses was a *P*-value of ≤ 0.01.

### Molecular docking

The molecular docking analysis was conducted employing AutoDock and AutodockTools-1.5.6 (developed by The Scripps Research Institute, La Jolla, CA, USA). The 3D structural data of the compounds were retrieved from the PubChem database (https://pubchem.ncbi.nlm.nih.gov/) and subsequently stored in the SDF format. We acquired the three-dimensional (3D) protein macromolecule structures from the Protein Data Bank (PDB) database, accessible at https://www.rcsb.org/, and retrieved them in the PDB file format. The removal of water molecules and ligands from the protein macromolecular structures was executed using PyMOL 3.8.5 software (Schrödinger, LLC, New York, NY, USA). The transformation of protein macromolecules and compounds into the PDBQT format was accomplished through the utilization of AutoDockTools 1.5.6 software. The compounds were subjected to molecular docking with the protein macromolecules, and the resultant docking scores were computed employing AutoDock Vina 1.1.2 (developed by the Center for Computational Structural Biology, The Scripps Research Institute) (Figure 1).

## Results

### Identification of active ingredients

Compounds derived from Xiao Ji were identified through a search conducted within the TCMSP database. Subsequently, 5 compounds were chosen based on specific criteria, specifically those with an OB ≥30% and a DL score ≥0.18. Acacetin (MOL001689), linarin (MOL001790), sitosterol (MOL000359), stigmasterol (MOL000449), and quercetin (MOL000098) were identified as the primary bioactive constituents of Xiao Ji, as presented in Table 1.

**Table 1.** The information of the 5 active components of setosum with the predicted OB ≥30% and DL ≥0.18

No.	Molecule ID	Molecule name	OB	DL	Structure
1	MOL001689	Acacetin	34.97	0.24	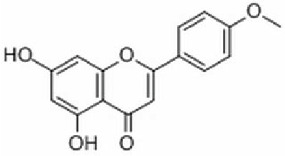
2	MOL000359	Sitosterol	36.91	0.75	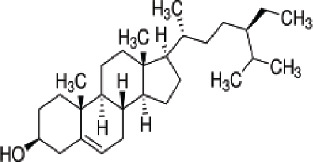
3	MOL000449	Stigmasterol	43.83	0.76	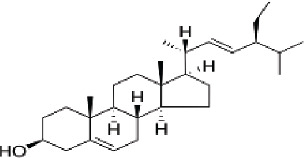
4	MOL000098	Quercetin	46.43	0.28	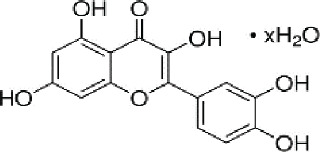
5	MOL001790	Linarin	39.84	0.71	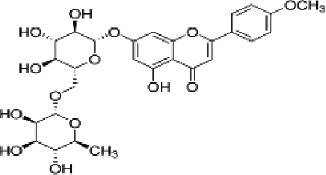

1 DL, drug likeness; OB, oral bioavailability.

### Identification and analysis of targets

**Figure 2.**
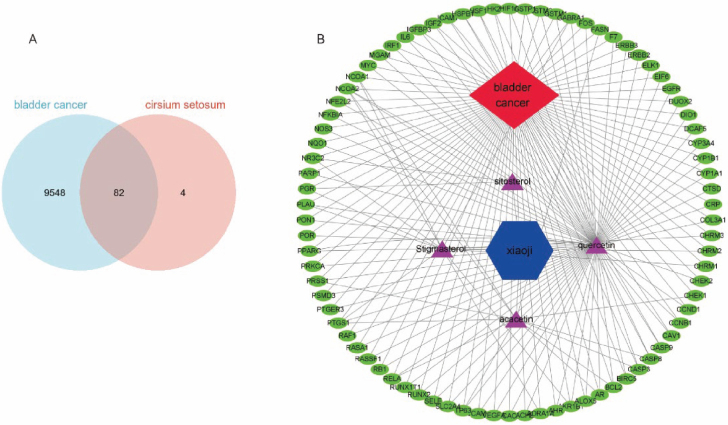
**(A)** The light green part represents the number of BC targets, and the light red part represents the number of Xiao Ji targets. **(B)** The purple hexagons represent Xiao Ji, the pink triangles represent the active ingredients of Xiao Ji, and the green ovals represent potential BC targets. BC, bladder cancer.

The targets associated with the 5 compounds were extracted from the TCMSP database, resulting in the identification of a total of 86 potential target proteins. In total, 9,548 genes associated with BC were obtained through retrieval from both the GeneCards and OMIM databases. The overlapping set of predicted targets for the 5 compounds and genes associated with BC were considered as potential therapeutic targets of Xiao Ji (Figure 2A).

### Construction and analysis of the I–T network

The I–T network, constructed using Cytoscape software, comprises 84 nodes and 101 edges (Figure 2B). I–T network analysis reveals that 4 out of the 5 compounds’ target proteins are associated with BC. The 4 compounds identified are acacetin, sitosterol, stigmasterol, and quercetin. The network consists of 80 oval nodes in green, representing the target genes, and 4 triangular nodes in pink, symbolizing the active ingredients. The 101 edges symbolize the interactions occurring between the ingredients and the target genes. The degree of connectivity of a node serves as an indicator of the node’s significance within the network. In the I–T network, quercetin exhibited the highest degree value, amounting to 74, followed by stigmasterol and acacetin, each with a degree of 12. This observation implies that quercetin holds a comparatively higher degree of significance within the network in comparison to the other active ingredients. Furthermore, it was observed that 14 genes (*AKR1B1, AR, BCL2, CASP3, CASP8, GABRA1, NCOA1, NR3C2, PGR, PLAU, PPARG, PRSS1, RELA*, and *TP63*) possessed two degrees, 1 gene (prostaglandinendoperoxide synthase 1 [*PTGS1*]) had three degrees, and 1 gene (nuclear receptor coactivator 2 [*NCOA2*]) exhibited four degrees. This pattern of connectivity suggests that these genes are the primary and central targets for the identified active ingredients.

### PPI network construction and analysis

**Figure 3.**
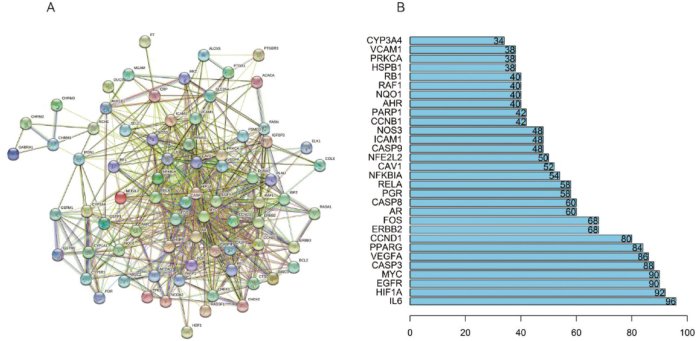
**(A)** PPI network of potential targets for BC is associated with Xiao Ji. The nodes represent the proteins and the edges represent their interactions. A protein with more edges is considered to play a more important role. **(B)** A bar plot of the top 30 target proteins, sorted according to degree values. The longer bar represents greater connection of the protein. BC, bladder cancer; PPI, protein–protein interaction.

The PPI network was assembled utilizing the STRING database to identify pivotal proteins. Applying a stringent confidence threshold of ≥0.4 and subsequently removing unconnected proteins, we acquired a total of 76 nodes and 2,632 edges within the PPI network (Figure 3A). In the network analysis, proteins with a higher number of edges are indicative of potentially playing more pivotal roles. Consequently, based on this network analysis, the critical genes identified included interleukin (IL)-6, hypoxia inducible factor 1 subunit alpha (HIF1A), epidermal growth factor receptor (EGFR), myelocytomatosis oncogene (MYC), caspase 3 (CASP3), vascular endothelial growth factor A (VEGFA), peroxisome proliferator-activated receptor gamma (PPARG), and cyclin D1 (CCND1) (Figure 3B).

### GO and pathway enrichment analyses

**Figure 4.**
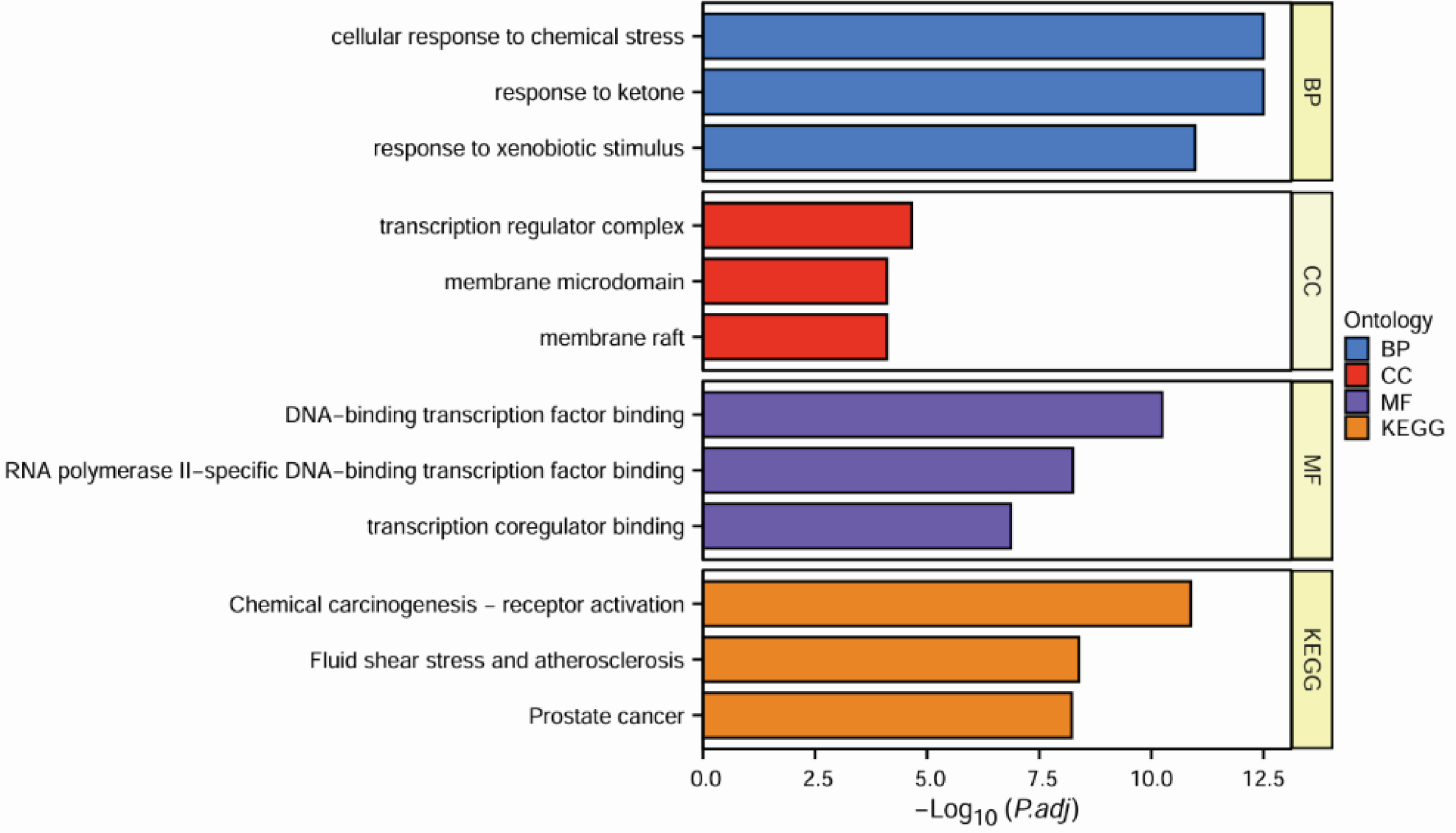
GO analysis and KEGG pathway enrichment of target genes for BC associated with Xiao Ji. BP, biological process; CC, cellular components; GO, gene ontology; KEGG, Kyoto encyclopedia of genes and genomes; MF, molecular function.

The 82 overlapping critical target genes were subsequently subjected to further analysis utilizing GO and KEGG to provide insights into the underlying mechanisms by which Xiao Ji operates in the treatment of BC. We identified a total of 1,208 significantly enriched GO terms and 107 pathways (adjusted *P* < 0.05; **Table S2** in the Supplementary Materials). In Figure 4, the visual representation highlights the foremost 3 significantly enriched GO terms within the biological process (BP), cellular components (CC), and molecular function (MF) categories, alongside the top 3 most enriched KEGG pathways. The involvement of GO terms such as “transcription factor binding,” “ubiquitin-like protein ligase binding,” and “ubiquitin protein ligase binding” have been identified in the pathogenesis of BC. Numerous pathways are linked with BC, including notable examples, such as “p53,” “apoptosis,” “HIF1A,” “tumor necrosis factor (TNF),” and “PI3K-Akt” pathway.

### Molecular docking

**Figure 5.**
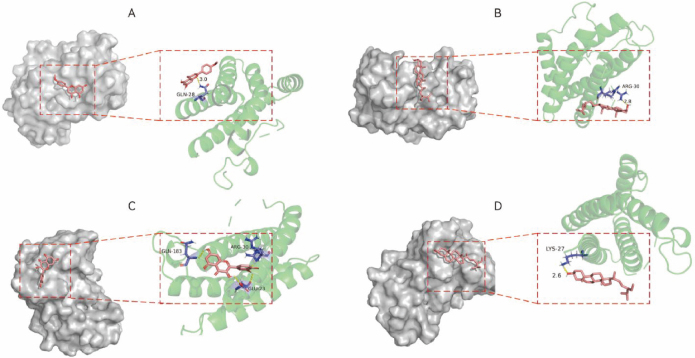
Part of molecular docking results. **(A)** Acacetin-IL6; **(B)** sitosterol-IL6; **(C)** quercetin-IL6; **(D)** stigmasterol-IL6. The compound structures are shown as red sticks, the protein structures are shown as green ribbons, and the interacting residues are presented as purple and yellow lines. IL-6, interleukin-6.

Four candidate target genes, distinguished by their elevated node scores in the network analysis, were subsequently chosen for molecular docking experiments with the 4 active compounds of Xiao Ji. The docking scores are documented in Table 2. A lower docking score is indicative of a more robust binding affinity between the ingredient and its target. The binding energies of all compounds, except quercetin, to the 3 pivotal target genes (*IL-6, EGFR*, and *MYC*) were found to be ≤–4 kJ/mol. This observation suggests that these ingredients exhibit a strong and tight binding affinity with the critical proteins, as depicted in Figure 5.

**Table 2.** The docking affinity and interactions of compounds binding to key targets

Molecule ID	Molecule name	Affinity (kcal/mol)			
		IL-6	EGFR	MYC	HIF1A
MOL001689	Acacetin	–4.12	–4.0	–4.37	–3.13
MOL000359	Sitosterol	–4.37	–4.54	–5.1	–3.71
MOL000449	Stigmasterol	–4.36	–4.68	–4.63	–3.93
MOL000098	Quercetin	–2.16	3.64	–3.63	–1.32

1 EGFR, epidermal growth factor receptor; HIF1A, hypoxia-inducible factor 1 subunit alpha; IL-6, interleukin-6; MYC, myelocytomatosis oncogene.

## Discussion

As the most prevalent malignancy of the urinary system, BC is associated with high rates of recurrence, progression, and mortality. Though immunotherapy has made great successes in BC treatment, only a small proportion of patients benefit from the treatment [15]. TCM stands as a significant reservoir for the development of innovative anticancer pharmaceuticals. Given the historical utilization of Xiao Ji in TCM for the treatment of hematuria, we applied network pharmacology as an investigative approach to ascertain its potential anti-BC properties.

Through network pharmacological analysis, we conducted a screening process, which led to the identification of 5 compounds from Xiao Ji. Four out of these five compounds were found to be associated with 82 target genes that were commonly shared between these compounds and genes related to BC. The 4 compounds subjected to the screening process were identified as quercetin, acacetin, stigmasterol, and sitosterol. Quercetin exhibited associations with 74 out of the total 80 screened targets, suggesting its prominence as the primary component of Xiao Ji in its anti-BC properties. Quercetin has the capability to influence multiple signaling pathways, including the PI3K/Akt/mTOR and MAPK/ERK1/2 pathways, as documented in prior research [16], Moreover, it has demonstrated anti-tumor properties in various cancer types, such as ovarian cancer [17], esophageal cancer [18], and breast cancer [19]. It exhibits the capacity to suppress the TAK1/JNK signaling pathway, thereby inducing apoptosis in BC cells [20]. Acacetin, functioning as an inhibitor of STAT3, manifests potent anticancer properties [21]. It possesses anti-carcinogenic properties in conditions such as lung cancer, prostate cancer, gastric carcinoma, and colorectal cancer [22]. Research investigations have provided evidence that stigmasterol exhibits anti-tumor properties across various malignancies, including but not limited to lung cancer, ovarian cancer, gastric cancer, and hepatic cancer. Its mechanism of action involves the inhibition of multiple signaling pathways, such as Nrf2 [23], Akt/mTOR [24], and JAK/STAT [25] signaling pathways. Sitosterol has demonstrated the ability to induce cell apoptosis in ovarian cancer [26], colon cancer [22], and hepatocellular carcinoma [27]. Furthermore, it exhibits anti-pancreatic cancer activities through the inhibition of epithelial-mesenchymal transition (EMT) [28].

In the I–T network, it is noteworthy that the NCOA2 was found to be a common target of all 4 active ingredients. NCOA2 is implicated in the processes of metastasis and castration resistance observed in prostate cancer [29]. It has been reported to promote the proliferation of human breast cancer cells through the MAPK/ERK pathway [30]. Conversely, the knockdown of NCOA2 has been shown to inhibit the proliferation, metastasis, and invasion of gastric cancer cells, primarily via the Wnt signaling pathway [31]. Moreover, *NCOA2* gene fusions have been identified in multiple cancer types, including spindle cell sarcomas [32], leukemia [33], rhabdomyosarcoma [34], and uterine sarcoma [35]. On the contrary, PTGS1 is implicated in angiogenesis [36], and inhibiting PTGS1 has demonstrated antiproliferative effects on BC cells [37].

PPI network analysis revealed that IL-6 exhibited the highest degree value, signifying its central role as a hub gene. This hub gene, IL-6, assumes pivotal functions in the pathogenesis and progression of various malignancies [38–40]. The upregulation of IL-6 in BC is associated with an advanced clinical stage, heightened recurrence rates, and unfavorable survival outcomes [41]. It can facilitate the progression of MIBC by augmenting the immunosuppressive capabilities of myeloid-derived suppressor cells [42]. Elevated serum levels of IL-6 were correlated with reduced recurrence-free survival in patients with NMIBC following intravesical gemcitabine therapy [43]. Hence, IL-6 represents one of the pivotal pathways through which Xiao Ji exerts its anti-BC effects. The expression of HIF1A is typically induced in hypoxic environments, leading to its capacity to trigger autophagy and augment cisplatin resistance in BC [44]. The EGFR signaling pathway assumes a pivotal role in oncogenesis and the progression of cancer, with its hyperactivity exhibiting a correlation with advanced stages and diminished survival rates in BC [45]. As a transcriptional regulator, MYC is implicated in the initiation and progression of various malignancies [46]. It related to drug resistance in BC [47]. CASP3 is a pivotal enzyme involved in cellular apoptosis. Its overexpression has been associated with DNA methylation and serves as an indicator of poor prognosis in BC [48]. VEGFA exhibits the capacity to facilitate tumor growth and metastasis through the augmentation of angiogenesis, establishing its status as a crucial biomarker in BC [49]. PPARG mutations frequently occur in BC, and a decreased expression of PPARG may potentially facilitate the development of bladder tumors [50]. CCND1, a pivotal cell cycle regulator, exhibits overexpression in various cancer types and is correlated with an unfavorable prognosis in BC [51, 52].

According to the GO and KEEG enrichment analyses, Xiao Ji demonstrates its potential to manifest anticancer effects on BC through a spectrum of diverse biological processes and intricate signaling pathways. Notably, some of the identified GO terms, such as “wound healing,” “response to oxidative stress,” “response to hypoxia,” “transcription regulator complex,” “membrane raft,” “RNA polymerase II transcription regulator complex,” “DNA-binding transcription factor binding,” “ubiquitin-like protein ligase binding,” “ubiquitin protein ligase binding,” “ubiquitin protein ligase binding,” “transcription factor binding,” and “ubiquitin-like protein ligase binding,” exhibited associations with the pathogenesis of cancer.

There is a strong association between wound healing and cancer. During the wound healing process, keratinocytes exhibit migratory and excessively proliferative behaviors similar to those observed in the cellular behaviors of tumor occurrence and metastasis [53]. The major pathways associated with wound healing are also highly active in cancer [54]. Hypoxia is commonly observed in the microenvironment of solid tumors [55]. The hypoxic microenvironment facilitates abnormal angiogenesis, thereby contributing to tumor progression and drug resistance [56]. Additionally, hypoxia promotes the expression of HIF1A, further stimulating EMT and consequently enhancing tumor invasion and resistance to chemotherapy [57]. Aberrant transcriptional regulation is one of the fundamental characteristics of tumors [58]. Sustained proliferation and evasion of apoptosis in tumors both rely on transcriptional regulation [59]. RNA polymerase II transcribes protein-coding genes in the eukaryotic genome, and its functionality is contingent upon interactions with a range of proteins to modulate its activity. Various factors are involved in regulating RNA polymerase II initiation, pausing, and elongation [60]. Changes in the regulation of gene transcription by RNA polymerase II form the basis of many diseases, particularly in cancer, where they become targets for therapeutic intervention in certain tumors [61]. Membrane rafts, enriched in cholesterol and proteins, play crucial roles in cellular signal transduction pathways [62]. Cholesterol regulates membrane fluidity, and its integration with membrane rafts influences the expression of pathways such as PI3K/AKT and Hh, thus playing a significant role in the pathogenesis and metastasis of prostate cancer [53]. The dysregulation of DNA-binding proteins is implicated in many human cancers [63]. Some DNA-binding proteins such as heterogeneous nuclear ribonucleoprotein L (HNRNPL), heterogeneous nuclear ribonucleoprotein U (HNRPU), and interleukin enhancer binding factor 2 (ILF2) are closely associated with endometrial cancer [64]. The DNA-binding protein chromodomain helicase DNA binding protein 1 like (CHD1L) is overexpressed in various solid tumors and promotes tumor progression in colorectal cancer by facilitating G1/S phase transition and inhibiting apoptosis [65], while in breast cancer, it promotes tumor invasion and metastasis through the PI3K/Akt/ARK5/mTOR/MMP pathway [66]. Ubiquitin ligases participate in various physiological processes within cells by regulating the ubiquitination of proteins. They are frequently dysregulated in human tumors, playing crucial roles in processes related to cancer initiation and progression, such as regulating apoptosis, cellular metabolism, and tumor angiogenesis [67, 68]. Aberrant expression of ubiquitination-related proteins leads to excessive degradation of tumor suppressor protein p53, affecting DNA repair and resulting in poor prognosis for cancer patients [69]. Ubiquitin ligases impact cell proliferation through multiple pathways such as the PI3K-AKT pathway and ERK signaling, leading to excessive cell proliferation and tumor formation [70, 71].

Several signaling pathways, including the “p53,” “apoptosis,” “HIF-1,” “TNF,” and “PI3K-Akt” signaling pathway, have been found to be intricately associated with BC. P53 mutations result in uncontrolled cell proliferation, contributing to carcinogenesis [72]. Aberrations in the P53 signaling pathway are prevalent in the majority of human cancers [73], and these anomalies are linked to the recurrence and progression of BC [74]. The HIF-1 signaling pathway assumes a pivotal role in the cellular response to hypoxia, often resulting from the rapid proliferation of cancer cells [75]. It is linked to the pathogenesis of various human diseases, including cancer. In the context of BC, the HIF-1 pathway potentially enhances glucose metabolism and emerges as a prospective therapeutic target [76]. TNF signaling functions in a variety of physiological and disease processes such as immunity, inflammation, sepsis, neurodegenerative diseases, multiple sclerosis, and tumor [77]. It assumes a pivotal role in oncogenesis and cancer immunotherapy, where the upregulation of TNFα (Tumor Necrosis Factor-alpha)-dependent pathways contributes to the advancement of BC to more advanced stages [78]. The PI3K-Akt signaling pathway holds a paramount position in the initiation and progression of cancer [79]. It fosters the migration and invasiveness of BC cells by instigating EMT and is recognized as a significant therapeutic target [80]. Therefore, the PI3K/AKT pathway may be a major therapeutic target of the Xiao Ji in treating BC.

We selected 4 pivotal genes from the PPI network and conducted molecular docking with the 4 active compounds derived from Xiao Ji. The results of molecular docking demonstrated a strong affinity between 3 active ingredients (acacetin, sitosterol, and stigmasterol) of Xiao Ji and the 3 key target proteins (IL-6, EGFR, and MYC). This suggests that Xiao Ji may potentially treat BC by suppressing IL-6, EGFR, and MYC.

This study has certain limitations. The pharmacological mechanisms underlying the therapeutic effects of Xiao Ji on BC were primarily predicted through data mining using a network pharmacology approach and subsequently validated solely through molecular docking techniques. This study did not account for the quantitative aspects of individual compounds and their potential interactions. Additionally, the metabolic pathways of these compounds within the human body and their specific impact on BC remain to be validated through further experimental research.

## Conclusion

We utilized a combined approach involving network pharmacology and molecular docking to elucidate the potential mechanism of action of Xiao Ji against BC. We have unveiled that 4 active components of Xiaoji, namely acacetin, sitosterol, stigmasterol, and quercetin, play pivotal roles in the treatment of BC by exerting their influence on IL-6, EGFR, and MYC. Furthermore, Xiao Ji demonstrates the capacity to modulate various signaling pathways, encompassing “p53,” “apoptosis,” “HIF-1,” “TNF,” and the “PI3K-Akt” pathway, serving as a potential therapeutic strategy for the management of BC. Ultimately, our molecular docking analyses substantiated the robust binding affinities of the 3 primary active compounds contained in Xiao Ji (acacetin, sitosterol, and stigmasterol) with IL-6, EGFR, and MYC. Nevertheless, experimental validation is imperative to corroborate these theoretical predictions.
